# How the preoperative ultrasound examination and BFI of the cervical lymph nodes modify the therapeutic treatment in patients with papillary thyroid cancer

**DOI:** 10.1186/1471-2482-13-S2-S52

**Published:** 2013-10-08

**Authors:** Giuseppina Napolitano, Antonio Romeo, Gianfranco Vallone, Michele Rossi, Luca Cagini, Gabriele Antinolfi, Mario Vitale, Luca Brunese, Eugenio Annibale Genovese

**Affiliations:** 1 Department of Health Science, University of Molise, Italy; 2 Department of Radiology, University of Naples "Federico II", Italy; 3 Azienda Ospedaliera Sant'Andrea, Department of Radiology, Rome, Italy; 4 Thoracic Surgery Unit, University of Perugia Medical School, Perugia, Italy; 5 Department of Medicine and Surgery, University of Salerno, Baronissi (SA), Italy; 6 Department of Radiology, University of Cagliari, Cagliari, Italy

## Abstract

**Background:**

Ultrasound is considered the best diagnostic method for the detection of metastatic cervical lymph nodes (LNs) in patients with papillary thyroid carcinoma (PTC). According to current guidelines, all patients undergoing thyroidectomy for malignancy should undergo preoperative neck ultrasound of the thyroid and central and lateral neck LNs, followed by fine needle aspiration of suspicious LNs. Cervical LN involvement determenes the extent of surgery. Complete surgical resection disease at the initial operation decreases likelihood of future surgery for recurrent disease and may impact survival. We use a new technique, B-flow imaging (BFI), recently used for evaluation of thyroid nodules, to estimate the presence of BFI twinkling signs (BFI-TS), within metastatic LNs in patients with PTC.

**Methods:**

Between September 2006 and December 2012, 304 patients with known PTC were examined for preoperative sonographic evaluation with gray-scale US, color Doppler US and BFI. Only 157 with at least one metastatic LN were included in our study. All patients included underwent surgery, and the final diagnosis was based on the results of histologic examination of the resected specimens. The following LN characteristics were evaluated: LN shape, abnormal echogenicity, the absent of hilum, calcifications, cystic appearance, peripheral vascularization and the presence of BFI-TS.

**Results:**

A total of 767 LNs were analyzed. 329 out of 767 were metastatic, according to the histopathologic findings. BFI-TS, showed 99.5% specificity and 81,5% sensitivity. We detected BFI-TS in 6 metastatic LNs that were negative to the other conventional US features.

**Conclusions:**

Our results indicate that the BFI-TS has a diagnostic accuracy higher than the other conventional sonographic signs. Our findings suggest that BFI can be helpful in the selection of suspicious neck LNs that should be examined at cytologic examination or open biopsy for accurate preoperative staging and individual therapy selection.

## Background

Several studies have shown that ultrasonography has higher sensitivity than palpation and the other diagnostic methods for the detection of cervical metastatic lymph nodes (LNs) in patients with papillary thyroid cancer (PTC) [1]. According to current guidelines, all patients undergoing thyroidectomy for malignancy should undergo preoperative neck ultrasound of the thyroid and central and lateral neck LNs, followed by fine needle aspiration (FNA) of suspicious LNs [2]. This practice has been widely accepted and supported by leading centers [3-9] and has been shown to change the surgical procedure performed in 39% of thyroid cancer patients[10-15]. The extent of surgery is determined by LN involvement of the central and lateral neck. Lateral neck disease discovered preoperatively leads to more aggressive surgery, which includes compartment-oriented LN dissection. Complete surgical resection disease at the initial operation decreases likelihood of future surgery for recurrent disease and may impact survival [2] Despite this; the rate of reoperation for early recurrences remains high. Technological advancements have allowed the identification of a new ultrasound technique the B-flow imaging (BFI) that has recently been used to evaluate thyroid nodules [12,13]. BFI can identify a new sign (the twinkling sign; BFI-TS) in "suspect" PTC nodules, which appeared to be generated by microcalcifications and colloid crystals and increase the US accuracy in identification of malignant nodules [14]. The same features described in thyroid nodules are also present in LN metastases. The aim of our study was to evaluate the BFI-TS accuracy to identify metastatic LNs compared to conventional ultrasound alone in the preoperative phase to reduce the risk of re-operation.

## Materials and methods

### Patients

Between September 2006 and December 2012, 304 patients with known PTC were examined at our institution for preoperative sonographic evaluation with gray-scale ultrasonography (US), color Doppler US and BFI-TS. 157 patients with suspicious metastatic cervical LN at US examination underwent FNAB for cytology and thyroglobulin determination in the aspirate fluid. Only 109 patients (38 men, 71 women; mean age 54 years range, 25-77 years) with at least one metastatic LN were included in our study. All these patients underwent surgery, and the final diagnosis was based on the results of histologic examination of the resected specimens. The mean interval between sonographic examination and surgery was 5.2 days (range, 1 -17 days). The study was conducted at the Department of Radiology and Endocrinology of the University of Naples Federico II and at the Department of Endocrinology of the Second University of Naples, according to the principles of the Declaration of Helsinki and approved by the Ethics Committee of the University of Molise. Written informed consent was obtained from all subjects.

### US and cytological examinations

Sonographic, color flow Doppler (CFD), and BFI examinations were performed with a LOGIQ 9 system (GE Healthcare, Chalfont St Giles, England), a commercially available real-time sonography system, equipped with 5 to 14 MHz (M12L) and 2.5 to 7MHz (7L) linear array transducers. All examinations were performed by two blinded radiologists with 8 and 10 years of neck sonography experience separately and all data analysis was performed by another investigator. When results of the examiners were discordant, agreement was found by conjoint review of clips of the US examinations. The following US characteristics were recorded for each nodule: round shape (ratio of short axis to long axis > 0.5), absence of echogenic hilum, abnormal ecogenicity of LN, calcification, cystic change and a peripheral color Doppler pattern. The location (levels I-VI) of all cervical LNs were recorded, based on the American Joint Committee on Cancer and the American Academy of Otolaryngology-Head and Neck Surgery nodal classification [15-17]. BFI was performed at 10 MHz (M12L) and 7 MHz (7L) with the BFI capability at the level of the LNs. PRI was set at 3. BFI gain was not fixed and was adjusted to allow a better visualization of the signs. This technique focuses on high flow, with suppression of the tissue signal. BFI images were used to evaluate the presence or the absence of the signs. The BFI-TS is a rapidly flashing white light behind such stationary objects as microcalcifications and colloidal crystals. The sign was considered positive whe n at least a twinkling was present in the LNs examined and repeatable over time. After the US features were assessed, patients underwent a cytological evaluation. US-guided FNA was simultaneously performed by an endocrinologist, a radiologist and pathologist. Physicians were highly experienced in carrying out US-guided FNA using 27- and 22-gauge needles; the technique used is described elsewhere [18,19]. After collection of the cytology samples, each FNAB needle was washed with 0.1-0.5 ml of normal saline. Thyroglobulin was measured in fine needle washouts using an immunoradiometric assay (IRMA-DYNO test Tg-plus, BRAHMS Diagnostic GmbH, Berlin, Germany). When the measured FNAB-Tg level was greater than the serum Tg level, we deemed the LN positive for metastasis from PTC.

### Surgery and histologic examination

All patients underwent thyroidectomy and ipsi- or bilateral modified radical neck dissection to include levels II-V. All possible measurements were taken to ensure an accurate one-to-one comparison between the LNs that were imaged and those that were removed during surgery. After US examination, the location of each LNs was mapped with respect to the surrounding anatomic structures (i.e., trachea, main vessels, and sternocleidomastoid muscle) and plotted on the sketched diagram of the neck. Surgeons were assisted by a radiologist for correlation of the LN location seen on the US images with the LNs seen in the lymphadenectomy specimens. After being resected, each LN specimen was fixed in 10% formalin, embedded in paraffin, cut into thin slices, and stained with standard hematoxylin-eosin. During histologic examination, two or three histologic slices per LN were examined. The final diagnosis of metastatic LN involvement was made by a pathologist who had 15 years experience in diagnosing histologic cervical LN. Complete versus incomplete metastatic involvement and the presence of necrosis and/or calcifications were also investigated.

### US and pathology correlation

To match each LN found at pathological examination to the corresponding node on US, we took into account its location, shape and size. Only LNs that were unequivocally matched between US and pathology were taken into account. Multiple LNs at a given neck level on US were taken into account only if all LNs of the compartment were either benign or malignant.

### Statistical analyses

Qualitative variables were compared by using the χ2test. The BFI characteristics of each LN were recorded separately and processed blindly for statistical evaluation. The unit of analysis was each LN rather than each patient. The value of each visual and qualitative criterion that showed the highest diagnostic accuracy in the distinction between benign and metastatic lymph nodes was selected as the cut off value. For each criterion examined, the sensitivity, specificity, positive and negative predictive values, and overall accuracy in the differentiation between benign and metastatic LNs were calculated. Quantitative data are reported as means ± 1 standard deviation. Statistical significance was assumed when the p value was less than 0.05. The same analysis has been performed on the association between the BFI and each ultrasound parameter.

## Results

A total of 767 LNs were analyzed. Of these, 329 were metastatic while the remaining 438 were benign, as evaluated by histopathology. The diagnostic performance of each ultrasound finding evaluated in this study is shown in Table 1. Most ultrasound features had high specificity and positive predictive value (PPV) but low sensitivity and negative predictive value (NPV). The only sonographic characteristic with high specificity and sensitivity was the BFI-TS. The BFI-TS was positive in all LNs with microcalcifications at US examination (118 LNs) and in 189 LNs (all metastatic) in which microcalcifications were not evident at US. Two LNs positive at the BFI-TS and with calcifications at US was found to be a tuberculous nodes after treatment with intranodal macrocalcifications at histological examination.

**Table 1:** The diagnostic performance of sonographic criteria for metastatic lymph nodes in 109 patients with papillary thyroid cancer.

US features	Total lymph nodes 767	Metastatic lymph nodes 329	Sensitivity (%)	Specificity (%)	P	PPV	NPV
Round shape Short to long Axis (diameter ratio > 0.5)	235	173	52.6	85.8	*Χ^2 ^*130.5 P 0.0000	73.6	70.7

Abnormal echogenicity	371	271	82.4	77.2	*Χ^2 ^*366.5 P 0.0000	73	85.4

Absent Hilum	512	304	92.4	52.5	*Χ^2 ^*170.8 P 0.0000	59.4	90.2

Calcification	120	118	35.9	99.5	*Χ^2 ^*178.5 P 0.0000	98.3	67.4

Cystic change	79	79	24	100	*Χ^2 ^*117.2 P 0.0000	100	63.7

Peripheral vascularity	313	163	49.5	65.8	*Χ^2 ^*18.2 P 0.0000	52.1	63.4

BFI-TS	309	307	81.5	99.5	*Χ^2 ^*673.4 P 0.0000	99.3	95.2


US, Ultrasound; LNs lymph nodes ; PPV, Positive predictive value; NPV, Negative predictive value; BFI-TS, B-Flow imaging twinkling s ign.

## Discussion

**Figure 1:**
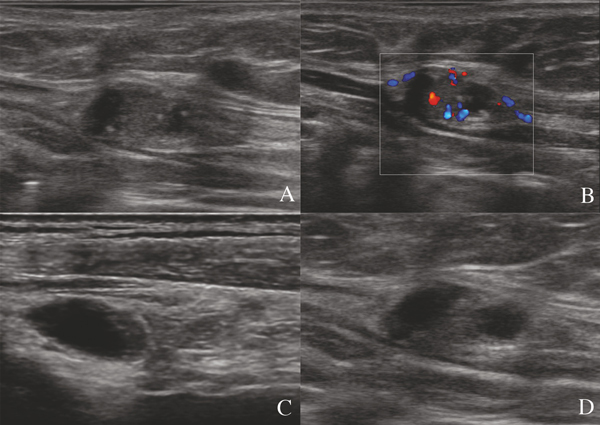
**Metastatic lymph nodes at gray-scale examination in patients with papillary thyroid cancer. Absence of echogenic hilum (A, B, C, D), abnormal echogenicity (A, B, C, D), calcifications (A, B), cystic change (C), abnormal vascolarization (B)**.

Ultrasound is the recommended imaging modality for pre-operative staging of PTC [2]. Appropriate staging is important not only for predicting outcome but for directing surgical resection. Radical resection of disease in the neck during the initial surgery is the best way to prevent reoperation. In various studies, the percentage of persistent or recurrent metastatic disease detected during the follow-up period was found to be 20-28% [20]. Much has been published on the accuracy of ultrasound for staging of the lateral neck in PTC [3-9]. The specificity reported varies from 85 to 90% [21-24]. A variety of diagnostic criteria have been reported to be useful for the distinction between benign and metastatic LNs. Metastatic LNs tend to be round, hypoechoic, and hypervascularized with a loss of hilar architecture and they may also demonstrate specific features such as hyperechoic punctuations or microcalcifications and cystic appearance, in differentiated thyroid cancer (Figure 1). In the present study, LN round shape had an excellent specificity (85.8%) but low sensitivity (52,6%). This can be explained by a partial or initial involvement of LN does not change the shape. In addition, some normal LNs may be rounded, especially in the parotid and submandibular regions [22].

The sonographic sign with higher sensitivity (92,4%) is the absence of echogenic hilum, but with low specificity (52,5%). The presence of a hyperechoic hilum of the nodes is usually considered a strong diagnostic criterion for benign LNs [24]. It has been reported that 84%-92% of benign nodes but less than 5% of metastatic nodes have a hyperechoic hilum [24]. The absence of a fatty hilum is often seen in normal individuals, especially in young subjects and in LNs located in level V [25]. Differently, abnormal LN echogenicity had both high sensitivity and specificity (respectively 82,4% and 77,2%). Calcification is a specific sign but not sensitive criterion. Calcification in metastatic LNs is characteristic of PTC but generally rare. In our study, nodal calcifications were detected in only 118 of the 329 metastatic LNs. Similarly, cystic appearance had a very high specificity (100%) and a low sensitivity (24%). All LNs with hyperechoic punctuations or a cystic appearance in a patient with PTC should be considered as malignant. Assessment of nodal vascularity at color Doppler US is another diagnostic criterion for metastatic LNs. It has been noted that benign LNs tend to show hilar vascularity or to appear avascular [26]. In contrast, metastatic nodes tend to have peripheral or mixed (both peripheral and hilar) vascularity [27-34]. In our study, color Doppler US vascularity had intermediate specificity (65,8%) but low sensitivity (49,5%). These findings could reflect the high differentiation of PTC and the reduced tendency to neoangiogenes is.

**Figure 2:**
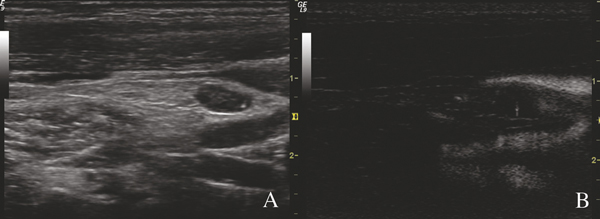
**Metastatic lymph nodes at B-mode and BFI examination in patients with papillary thyroid cancer. The lymph node presents microcalcification and a single BFI-TS in the same place**.

The BFI-TS had a higher specificity and sensitivity (respectively 99.5% and 81.5%) than conventional US features (Figure 2). The BFI-TS was positive in all LNs with calcification on US (118 LNs) and in 189 LNs (all metastatic) in which calcifications were not identified on US. The BFI-TS identified significantly more microcalcifications than B- mode US and it also identified highly reflective and non-calcified structures such as colloidal crystals. This is confirmed by the histological findings of micro-calcifications and colloidal crystals in the sites of BFI-TS. We detected BFI-TS in 6 metastatic LNs that were negative to the other conventional US features. Given its high specificity (99.7%) BFI-TS identifies better suspicious LNs that should be re-evaluated by surgery or US-guided FNAC [35-39]. The BFI is an ultrasound technique that integrates conventional ultrasound but it does not replace it. When a LN presents at least suspect ultrasound signs, it has to be studied also with the BFI since the positivity to BFI-TS gives evidence of its metastatic involvement with high diagnostic accuracy. The techniques has several limits, namely, it can be affected by the pulsatility of the main neck vessel and by the deep places of examined LNs. These limits could explain the missed detection of 22 LNs (7%) that were metastatic at histological examination. The other limit is the presence of non-metastatic LN calcifications; in fact the BFI- TS was false-positive only in two LNs with calcifications deriving from tuberculosis [40-43]. Overall, our results indicate that this technique can be applied to studies of cervical nodes in patients with PTC and that its sensitivity and specificity is higher than those of traditional US diagnostic techniques.

## Conclusions

BFI is a promising imaging technique that can help in the differentiation of benign and metastatic neck LNs in patients with PTC. Our findings suggest that BFI, in addition to conventional ultrasound, can be helpful in the selection of suspicious neck LNs that should be examined cytologically or with open biopsy for accurate preoperative staging and individual therapy selection. A dedicated cervical US that includes nodal levels II-VI should be performed to detect non palpable LN metastases in patients undergoing surgical evaluation. This practice has been shown to change the surgical procedure performed in 6 patients with PTC. However, longitudinal studies on a large population are required to verify the efficacy of BFI in the diagnosis of metastatic LNs.

## Abbreviations

PTC, papillary thyroid cancer; LNs, lymph nodes; US, ultrasonography; BFI, B-flow imaging, BFI-TS, twinkling signs; FNA, fine needle aspiration.

## Competing interests

The authors declare that they have no competing interests.

## Authors' contributions

GN: conceived the study, carried out the examinations, analyzed and interpreted the data, drafted the manuscript. AR: conceived the study, carried out the examinations, analyzed and interpreted the data. GV: conceived the study, carried out the examinations, analyzed and interpreted the data. MR: critically revised the manuscript. LC: critically revised the manuscript. GA: critically revised the manuscript. MV: critically revised the manuscript. LB: conceived the study, analyzed and interpreted the data, critically revised the drafted manuscript. EAG: conceived the study, analyzed and interpreted the data, critically revised the drafted manuscript. All authors read and approved the final manuscript.

## Authors' information

GN: Post-Doctoral Fellow in Radiology at University of Naples "Federico II". AR: Post- Doctoral Fellow in Radiology at University of Naples "Federico II". GV: Assistant Professor of Radiology at University of Naples "Federico II". MR: Assistant Professor of Radiology at University of Rome "Sapienza". LC: Assistant Professor of Radiology at University of Perugia. GA: PhD Student in Radiology at Second University of Naples. MV: Associate Professor of Endocrinology at University of Salerno. LB: Full Professor of Radiology at University of Molise. EAG: Associate Professor of Radiology at University of Cagliari.

## Declarations

Funding for this article has come from a company within the health sector.

This article has been published as part of *BMC Surgery *Volume 13 Supplement 2, 2013: Proceedings from the 26th National Congress of the Italian Society of Geriatric Surgery. The full contents of the supplement are available online at http://www.biomedcentral.com/bmcsurg/supplements/13/S2
